# Assessing in-hospital mortality risk in ICU lung cancer patients using machine learning: An analysis based on the MIMIC-IV database

**DOI:** 10.1371/journal.pone.0341259

**Published:** 2026-01-22

**Authors:** Jianwei Wang, Lizhen Lin, Li-ping Qiu, Li-lan Zheng, Lu-xi Wu, Hui Lv, Haihua Xie

**Affiliations:** 1 Department of Clinical Laboratory, Fuzhou University Affiliated Provincial Hospital, Fuzhou, China; 2 Department of Clinical Laboratory, Fuzhou Second General Hospital, Fuzhou, China; PLOS ONE, UNITED KINGDOM OF GREAT BRITAIN AND NORTHERN IRELAND

## Abstract

**Background:**

Patients with advanced lung cancer admitted to the intensive care unit (ICU) face a substantially elevated risk of in-hospital mortality. Early identification of high-risk individuals is essential to support timely clinical decision-making. This study aimed to develop and validate a predictive model using machine learning (ML) techniques to estimate in-hospital mortality in this patient population.

**Methods:**

Clinical data were obtained from the Medical Information Mart for Intensive Care-IV (MIMIC-IV) database. Feature selection was performed using least absolute shrinkage and selection operator (LASSO) regression, enabling the construction of eight ML models: logistic regression (LR), support vector machine (SVM), gradient boosting machine (GBM), artificial neural network (ANN), extreme gradient boosting (XGBoost), k-nearest neighbors (k-NN), adaptive boosting (AdaBoost), and random forest (RF). Model performance was assessed using the area under the receiver operating characteristic curve (AUC), as well as accuracy, sensitivity, specificity, and F1 score. Discrimination, calibration, and clinical utility were also evaluated. The final model incorporated 27 clinically interpretable variables, including not only established severity scores (e.g., SAPS II) but also dynamic treatment factors (e.g., vasopressin, mechanical ventilation duration) that reflect real-world ICU practice. SHAP analysis was employed to enhance interpretability, allowing clinicians to understand both the magnitude and directionality of key predictors—an improvement over black-box ML applications in prior studies.

**Results:**

Among the 1,755 patients included, 368 (21%) died during hospitalization in the training cohort.Notably, older individuals, particularly those of Caucasian descent, demonstrated a higher susceptibility to mortality during their hospital stay. Lasso regression revealed that 27 variables demonstrated a significant correlation with lung cancer, such as gender, hospital stay duration The XGBoost model achieved the highest predictive performance, achieving an accuracy of 0.783, an F1 score of 0.595, and an AUC of 0.865 (95% CI: 0.840–0.891)within the training cohort. The performance metrics for the test cohort reflected similar trends, with an accuracy of 0.719, an F1 score of 0.543, and an AUC of 0.790(95% CI: 0.741–0.840). Key predictors identified consistently across models (LR, SVM, ANN, and XGBoost) included hospital stay duration, Simplified Acute Physiology Score II (SAPS II), use of norepinephrine and vasopressin, prothrombin time (PT), mechanical ventilation duration, white blood cell count (WBC), and blood urea nitrogen (BUN). The SHAP summary plot further illustrated the direction and magnitude of influence for the top 15 predictors.

**Conclusion:**

The XGBoost-based model showed the best performance in predicting in-hospital mortality among critically ill lung cancer patients. Hospital stay duration and SAPS II score emerged as the most influential predictors,which can serve as the basis for a simplified clinical risk score. These findings may support early risk stratification and guide clinical decision-making in the ICU. The analysis, relying exclusively on internal divisions from MIMIC-IV, restricts the model’s generalizability and, consequently, its applicability in broader clinical contexts.

## Introduction

Lung cancer is a leading cause of cancer-related mortality worldwide, with a significant number of patients facing poor prognoses due to diagnoses made at advanced stages and inadequate treatment options [1–3]. The prognosis for lung cancer patients in the intensive care unit (ICU) is particularly bleak, with studies indicating in-hospital mortality rates that can be as high as 69% [4]. Moreover, identifying prognostic factors, such as performance status and biochemical markers, is essential for developing effective care strategies and prioritizing ICU admissions. Recent research has highlighted the increased mortality rates among lung cancer patients admitted to the ICU, thereby emphasizing the urgent need for reliable early prediction and risk stratification methods to identify high-risk individuals and improve clinical outcomes [5–7].By filling this knowledge gap, we can better allocate ICU resources, optimize treatment plans, and offer more accurate prognoses to patients and their families. Therefore, there is an immediate need for research that focuses specifically on predicting in – hospital mortality in this vulnerable patient population.

Machine learning (ML) offers a promising alternative due to its ability to analyze complex datasets, revealing patterns and relationships that may remain hidden in traditional statistical analyses [8,9]. This highlights the necessity for dependable predictive models that can accurately identify patients at increased risk of mortality during hospitalization. As an innovative form of artificial intelligence, ML has the potential to transform measurement results into relevant predictive models, particularly in oncology, aided by rapid advancements in large datasets and deep learning techniques. Recent findings demonstrate that ML has been effective in predicting lung cancer susceptibility, recurrence, survival rates, and the prognosis of malignant tumors [10–13]. However, there is a notable lack of data regarding in-hospital mortality risk prediction models utilizing ML approaches specifically for lung cancer patients in an ICU setting.

Previous studies may have only used a single or a few machine – learning models to predict the in – hospital mortality of critically ill lung cancer patients. In this study, eight different machine – learning models were constructed, including Logistic Regression (LR), Support Vector Machine (SVM), Gradient Boosting Machine (GBM), Artificial Neural Network (ANN), eXtreme Gradient Boosting (XGBoost), k – Nearest Neighbors (k – NN), Adaptive Boosting (AdaBoost), and Random Forest (RF). By comparing multiple models, it is possible to comprehensively evaluate the performance of different models in predicting the in – hospital mortality of this specific patient group, and thus select the optimal model.

Despite the high accuracy levels achieved by these ML models, the specific contributions of individual variables to their performance often remain unclear. This ambiguity limits the practical application of ML methodologies in clinical environments [14]. Shapley Additive ExPlanations (SHAP) provides a solution by integrating optimal credit allocation with localized interpretations, visually elucidating the importance of each variable within the model [15], thereby enhancing interpretability.

This study aims to utilize the extensive data available in the MIMIC-IV database to develop a robust ML model for predicting in-hospital mortality in lung cancer patients. The model will be validated using various algorithms, including logistic regression (LR), support vector machine (SVM), gradient boosting machine (GBM), artificial neural network (ANN), extreme gradient boosting (XGBoost), k-nearest neighbors (KNN), adaptive boosting (AdaBoost), and random forest (RF), thereby allowing a comprehensive assessment of its predictive capability. The identification of significant risk factors associated with in-hospital mortality will be prioritized, with the visual interpretation of the model using SHAP techniques to assist clinicians in identifying and intervening with high-risk populations.

## Methods

### Data source

This retrospective cohort study utilized data from the Medical Information Mart for Intensive Care-IV (MIMIC-IV, v2.2) (https://physionet.org/content/mimiciv/2.1/) [16,17], a clinical database that includes 730,141 ICU admissions from Beth Israel Deaconess Medical Center between 2008 and 2019, located in the United States. Since the study utilized non-identifiable, publicly available data, it received an exemption from informed consent requirements and Institutional Review Board (IRB) approval in accordance with ethical guidelines. All research procedures adhered to the ethical principles outlined in the 1964 Declaration of Helsinki and its subsequent amendments. Before data extraction, the research team completed the required web-based training modules and successfully passed the Protecting Human Research Participants certification exam, thereby obtaining authorized access to the MIMIC-IV database. A verbal notification was submitted to the institutional ethics committee, waiving the need for formal approval due to the retrospective, anonymized nature of the study.

### Cohort selection

**Fig 1:**
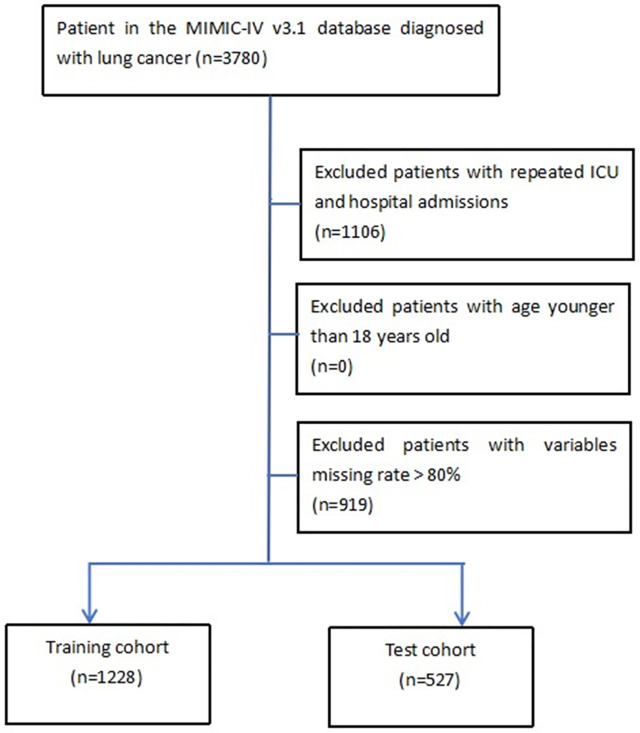
The flowchart of this study.

Individuals meeting any of the following criteria were excluded from the study: (1) those under 18 years of age at the time of their initial ICU admission; (2) patients with multiple ICU admissions; (3) cases with over 80% of personal data missing. We employed a random selection from the MIMIC-IV database to create both the training and test cohorts. Ultimately, this study included 1,288 patients in the training cohort and 527 patients in the test cohort, as depicted in the detailed flowchart presented in **Fig 1**.

### Data collection and outcomes

Data extraction from the database regarding patients admitted to the ICU within the first 24 hours was conducted using structured query language (SQL) in PostgreSQL. The variables considered in this investigation included the following categories: (1) Demographic information: gender, race, admission age, and weight; (2) Laboratory indicators: blood urea nitrogen (BUN), prothrombin time (PT), red blood cell count (RBC), red cell distribution width (RDW), partial thromboplastin time (PTT), chloride, anion gap, hemoglobin, white blood cell count (WBC), platelet count, creatinine, potassium, sodium, calcium; (3) Comorbidities: congestive heart failure, chronic pulmonary disease, peptic ulcer disease, diabetes, renal disease, Charlson comorbidity index, mild liver disease; (4) Vital signs: heart rate (HR), respiratory rate (RR), systolic blood pressure (SBP), diastolic blood pressure (DBP), temperature, percutaneous arterial oxygen saturation (SpO2); (5) Scoring systems: sequential organ failure assessment (SOFA) score, glasgow coma scale (GCS), acute physiology score III (APS III); (6) Interventions: continuous renal replacement therapy (CRRT), mechanical ventilation, vasopressin, losartan, epinephrine, dopamine, norepinephrine. For multiple measurements, the maximum and minimum values recorded on the first day were utilized, with the exception of SpO2. To reduce the impact of missing data on model development, the KNN Imputer method was employed to impute values for data with less than 30% missingness, whereas data with over 30% missingness were excluded from the analysis. The primary outcome of interest was in-hospital mortality.

### Statistical analysis

The Kolmogorov–Smirnov test was employed to analyze continuous variables. Given that all continuous variables demonstrated non-normal distributions, data were summarized using the median and interquartile range. To assess differences between groups, the Mann–Whitney U test was applied. Categorical variables were reported as percentages (%), and Pearson chi-squared tests were conducted to evaluate group differences. To address the class imbalance observed in the dependent variables, under-sampling techniques were implemented to readjust the dataset for balance. The sample data were partitioned into a training set and an internal validation set using fivefold cross-validation sampling. In scenarios involving numerous features, the LASSO technique was utilized for feature selection on the training set, which incorporates L1 regularization to select relevant features and reduce dimensionality by compressing coefficients, thereby identifying features with significant contributions whereas discarding redundant ones.

### Development of predictive models for in-hospital mortality

This research implemented eight ML algorithms: LR, SVM, GBM, ANN, XGBoost, KNN, AdaBoost, and RF to develop a predictive model for in-hospital mortality. Use one-hot encoding to handle categorical variables in LR, SVM, ANN, and KNN, while label encoding is recommended for GBM, XGBoost, AdaBoost, and RF.The variables selected through lasso regression were incorporated into the model. To ensure the model’s robustness, ten-fold cross-validation was utilized. Parameter tuning was conducted using grid search to identify the optimal tuning parameters for each algorithm. During the parameter adjustment phase, the model exhibiting the highest area under the curve (AUC) from the receiver operating characteristic (ROC) analysis was identified as the most effective model. The models were constructed using the training dataset, followed by validation of the best model using both internal and external validation datasets.

LASSO regression can effectively identify these key features with linear relationships and retain them. Subsequently, XGBoost can further explore the possible non – linear interaction effects between features on the basis of these selected features. In this way, the hybrid approach combines the advantages of both models, leveraging LASSO regression’s effective capture of linear relationships and XGBoost’s ability to mine non – linear information between features, thereby improving the overall performance of the model.

### Performance assessment

The evaluation of the predictive model’s performance was conducted utilizing the AUC of the ROC curve, as well as metrics such as sensitivity, specificity, F1 score, accuracy, and threshold values. Furthermore, decision curve analysis (DCA) and calibration curves were generated to illustrate the true clinical applicability of the model.

### SHAP analysis

To further investigate the positive or negative effects of the significant features identified for predicting in-hospital mortality, a SHAP analysis was executed using Python version 3.7.0. The SHAP value represents the predicted contribution of each feature within the dataset.

## Results

### Baseline characteristics

Following a thorough screening process, a cohort of 1,755 patients from the MIMIC-IV database was identified for inclusion in this study, of which 368 individuals (21%) died after admission to the ICU. As presented in **Table 1**, the baseline characteristics of the enrolled patients in the MIMIC-IV database were summarized. Notably, older individuals, particularly those of Caucasian descent, demonstrated a higher susceptibility to mortality during their hospital stay. These patients also experienced extended hospital stays following ICU admission, coupled with significantly elevated in-hospital mortality rates.

**Table 1:** Characteristic at baseline between survivors and non-survivors group in MIMIC- IV.

Variables	in-hospital mortality in lung cancer patients			P
	Total(n = 1755)	Survival(n = 1387)	Non-Survival(n = 368)	
Gender = M,n(%)	917 (52.3)	720 (51.9)	197 (53.5)	0.621
admission_Age (mean (SD))	69.94 (10.92)	69.54 (11.00)	71.44 (10.52)	0.003
Race = WHITE,n(%)	1176 (67.0)	963 (69.4)	213 (57.9)	<0.001
Laboratory values (mean (SD))				
INR	1.38 (0.78)	1.32 (0.67)	1.59 (1.06)	<0.001
BUN,mmol/L	22.89 (16.75)	21.28 (14.98)	29.15 (21.22)	<0.001
Potassium,mmol/L	4.23 (0.65)	4.20 (0.62)	4.35 (0.78)	<0.001
Sodium,mmol/L	137.21 (5.02)	137.33 (4.88)	136.75 (5.50)	0.053
Creatinine,mg/dl	1.05 (0.90)	1.02 (0.86)	1.20 (1.00)	0.001
Bilirubin_total,μmol/L	0.87 (1.78)	0.71 (0.94)	1.23 (2.89)	<0.001
ALT,U/L	83.00 (291.63)	69.50 (265.08)	115.60 (345.96)	0.038
ALP,U/L	129.32 (142.48)	115.66 (118.44)	162.00 (184.14)	<0.001
AST,U/L	118.56 (425.88)	93.02 (408.15)	179.54 (460.66)	0.007
LDH,mmol/L	626.98 (1421.48)	476.47 (896.24)	942.15 (2106.66)	<0.001
CK,U/L	380.98 (1028.67)	381.56 (855.23)	379.38 (1407.69)	0.985
PT,s	15.03 (7.44)	14.37 (5.81)	17.39 (11.23)	<0.001
RBC,109/L	3.62 (0.70)	3.66 (0.69)	3.45 (0.70)	<0.001
WBC,109/L	12.60 (7.99)	12.01 (6.79)	14.87 (11.24)	<0.001
Platelet,109/L	257.12 (130.04)	257.66 (124.80)	255.07 (148.79)	0.741
RDW,%	15.24 (2.22)	15.05 (2.13)	15.94 (2.42)	<0.001
Hemoglobin,g/L	10.64 (2.06)	10.80 (2.03)	10.00 (2.04)	<0.001
Hematocrit,%	32.75 (6.03)	33.12 (5.95)	31.32 (6.09)	<0.001
So2,%	87.26 (16.75)	89.10 (14.96)	84.21 (19.01)	0.005
Po2,mmHg	109.70 (88.01)	114.49 (88.38)	97.89 (86.11)	0.007
Ph	7.36 (0.09)	7.37 (0.08)	7.33 (0.12)	<0.001
PTT,s	35.65 (22.33)	35.21 (21.64)	37.24 (24.59)	0.151
Baseexcess,mmol/L	0.03 (5.16)	0.51 (4.58)	−1.14 (6.23)	<0.001
Albumin,g/dL	2.93 (0.61)	3.04 (0.59)	2.67 (0.56)	<0.001
Chloride,mmol/L	101.99 (5.80)	102.12 (5.62)	101.51 (6.47)	0.082
Aniongap,mEq/L	14.06 (3.71)	13.80 (3.35)	15.06 (4.73)	<0.001
Bicarbonate,mEq/L	23.91 (4.57)	24.19 (4.18)	22.85 (5.74)	<0.001
Calcium,mg/dL	8.52 (0.86)	8.54 (0.78)	8.42 (1.09)	0.025
Vital signs				
Weight,kg	75.23 (20.17)	76.01 (20.47)	72.77 (19.03)	0.053
Heart_rate,bpm	94.70 (21.39)	92.78 (20.68)	101.96 (22.45)	<0.001
SBP,mmHg	124.58 (24.03)	125.62 (23.96)	120.67 (23.91)	<0.001
DBP,mmHg	70.37 (17.90)	70.23 (17.48)	70.90 (19.42)	0.525
MBP,mmHg	85.00 (18.60)	85.25 (17.71)	84.07 (21.64)	0.281
resp_rate,bpm	21.03 (6.25)	20.58 (6.00)	22.76 (6.87)	<0.001
Temperature,°C	36.72 (0.61)	36.73 (0.58)	36.68 (0.74)	0.169
Spo2,%	95.97 (3.90)	96.12 (3.55)	95.38 (4.96)	0.001
Glucose,mg/dL	142.90 (61.19)	141.06 (59.84)	150.08 (65.77)	0.014
Comorbidities, n (%)				
Congestive_heart_failure	345 (19.7)	262 (18.9)	83 (22.6)	0.134
Chronic_pulmonary_disease	838 (47.7)	654 (47.2)	184 (50.0)	0.361
Peptic_ulcer_disease	38 (2.2)	27 (1.9)	11 (3.0)	0.308
Diabetes	388 (22.1)	303 (21.8)	85 (23.1)	0.657
Renal_disease	285 (16.2)	215 (15.5)	70 (19.0)	0.122
Charlson comorbidity	8.65 (2.79)	8.44 (2.77)	9.44 (2.74)	<0.001
Mild_liver_disease	97 (5.5)	66 (4.8)	31 (8.4)	0.009
Score system,points				
SOFA	1.11 (1.56)	0.95 (1.37)	1.73 (2.00)	<0.001
Sapsii	39.93 (12.50)	37.43 (10.53)	49.33 (14.72)	<0.001
GCS	14.55 (1.40)	14.62 (1.20)	14.29 (1.96)	<0.001
Ventilator,n(%)	1391 (79.3)	1081 (77.9)	310 (84.2)	0.01
Ventilator_hours,h	62.28 (83.37)	58.08 (80.77)	76.91 (90.49)	<0.001
CRRT n (%)	22 (1.3)	12 (0.9)	10 (2.7)	0.01
Vasopressin,n (%)	85 (4.8)	27 (1.9)	58 (15.8)	<0.001
Drug,n(%)				
Epinephrine_used	20 (1.1)	5 (0.4)	15 (4.1)	<0.001
Norepinephrine_used	244 (13.9)	123 (8.9)	121 (32.9)	<0.001
Dopamine_used	22 (1.3)	7 (0.5)	15 (4.1)	<0.001
Los hospital,d(mean (SD))	10.18 (8.99)	8.98 (6.57)	12.13 (10.38)	< 0.001
Los ICU,d(mean (SD))	3.59 (4.63)	3.40 (4.55)	4.31 (4.89)	0.001

Furthermore, patients categorized as Non-Survival exhibited elevated rates of Charlson comorbidity and mild liver disease. On the first day following admission to the ICU, Non-Survival patients were more likely to necessitate interventions, including the administration of vasopressors, CRRT, mechanical ventilation, and medications such as epinephrine, norepinephrine, and dopamine. Laboratory evaluations and vital sign measurements indicated that patients who did not survive demonstrated significantly lower levels of RBC, hemoglobin, SpO2, pH, base excess, albumin, anion gap, bicarbonate, and calcium. In contrast, these patients exhibited elevated concentrations of BUN, potassium, creatinine, total bilirubin, alanine aminotransferase (ALT), alkaline phosphatase (ALP), aspartate aminotransferase (AST), lactate dehydrogenase (LDH), PT, international normalized ratio (INR), WBC, RDW and the anion gap when compared to lung cancer patients who survived.

### Feature selection

**Fig 2:**
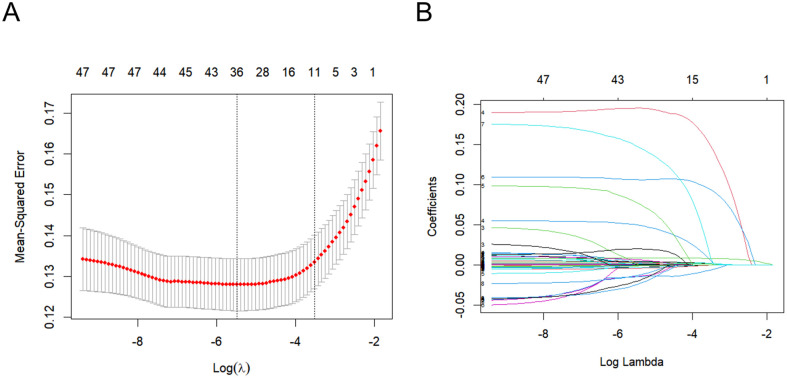
Lasso regression-based variable screening.

Lasso regression was employed to identify relevant features within the training dataset, with the characteristics of the variable coefficients illustrated in **Fig 2**. An iterative analysis employed a tenfold cross-validation approach. This analysis revealed that 27 variables demonstrated a significant correlation with lung cancer, including gender, hospital length of stay, age at admission, race, BUN, potassium, sodium, creatinine, PT, RBC, WBC, platelet count, RDW, hemoglobin, PTT, chloride, anion gap, calcium, heart_rate, SBP, DBP, resp_rate, temperature, SpO2, congestive heart failure, peptic ulcer disease, Charlson comorbidity index, mild liver disease, SOFA score, SAPS II score, GCS, ventilator hours, vasopressin usage, epinephrine usage, norepinephrine usage, and dopamine usage.

### Model performance comparisons

**Fig 3:**
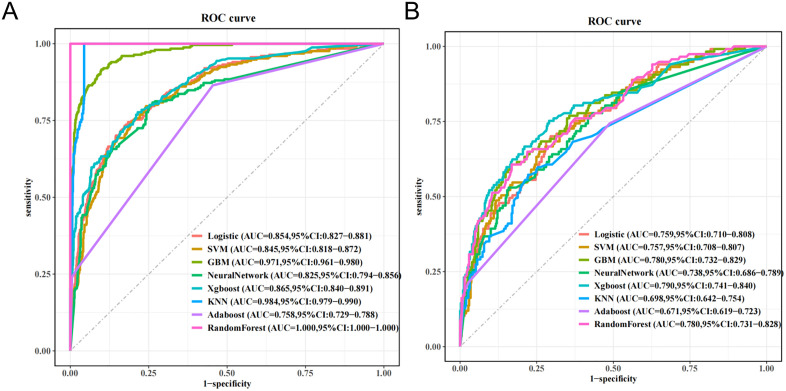
The performance of the eight in-hospital mortality predictive models.

In our study, we developed eight ML models to predict in-hospital mortality using a comprehensive set of features. The performance of these models is summarized in **Table 2**. Notably, the RF, KNN, and GBM models exhibited suboptimal predictive capabilities, as indicated by overfitting in the ROC curves. In contrast, the XGBoost model emerged as the most effective, achieving an accuracy of 0.783, an F1 score of 0.595, and an AUC of 0.865 within the training cohort. The performance metrics for the test cohort reflected similar trends, with an accuracy of 0.719, an F1 score of 0.543, and an AUC of 0.790 (refer to **Table 2).** Furthermore, we conducted a ROC analysis to validate the predictive capabilities of these eight models concerning in-hospital mortality. As illustrated in **Fig 3A and 3B**, the RF, KNN, and GBM models consistently demonstrated the least predictive power, whereas the XGBoost model exhibited superior performance across both the training and test cohorts.

**Table 2:** Performance of the prediction models using all features.

Model	Sensitivity	Specifcity	F1 score	Accuracy	Threshold	AUC	95%CI
Training cohort							
Logistic	0.745	0.816	0.605	0.801	0.218	0.854	0.827 − 0.881
SVM	0.797	0.758	0.582	0.766	0.168	0.845	0.818 − 0.872
GBM	0.916	0.901	0.796	0.904	0.230	0.971	0.961 − 0.980
NeuralNetwork	0.809	0.738	0.572	0.752	0.154	0.825	0.794 − 0.856
Xgboost	0.777	0.785	0.595	0.783	0.497	0.865	0.840 − 0.891
KNN	1	0.956	0.921	0.965	0.2947	0.984	0.979 − 0.990
SAdaboost	0.865	0.546	0.476	0.611	0.1697	0.758	0.729 − 0.788
RandomForest	1	1	1	1	0.435	1.000	1.000 − 1.000
Test cohort							
Logistic	0.701	0.705	0.513	0.704	0.158	0.759	0.710 − 0.808
SVM	0.65	0.741	0.508	0.721	0.171	0.757	0.708 − 0.807
GBM	0.607	0.834	0.555	0.784	0.228	0.780	0.732 − 0.829
NeuralNetwork	0.53	0.841	0.508	0.772	0.234	0.738	0.686 − 0.789
Xgboost	0.752	0.71	0.543	0.719	0.497	0.790	0.741 − 0.840
KNN	0.573	0.778	0.487	0.732	0.146	0.698	0.642 − 0.754
SAdaboost	0.744	0.512	0.431	0.564	0.169	0.671	0.619 − 0.723
RandomForest	0.607	0.832	0.553	0.782	0.283	0.780	0.731 − 0.828

**Fig 4:**
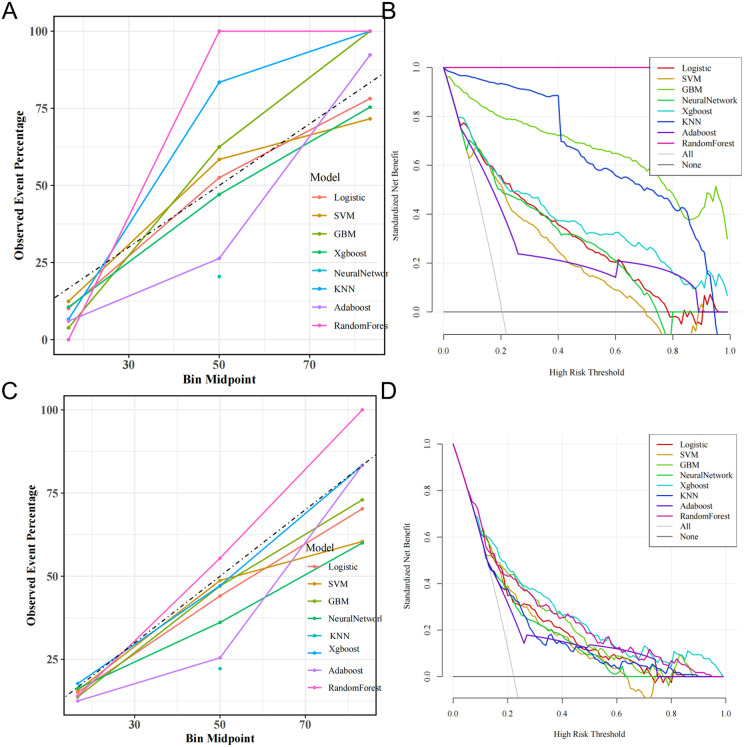
Calibration capability and clinical beneft of the model.

The calibration curves for all eight models are depicted in Fig 4A, C, providing essential insights into their predictive accuracy. Among the evaluated models, five—excluding the RF, KNN, and GBM—exhibited commendable agreement between predicted probabilities and actual observed results. Regarding clinical utility, all models, apart from the RF, KNN, and GBM, demonstrated significant net benefit across a broad spectrum of threshold probabilities. Notably, the XGBoost model provided the highest net benefit, establishing it as the most effective model for forecasting in-hospital mortality in lung cancer patients (Fig 4B, D).

### Feature importance analysis

**Fig 5:**
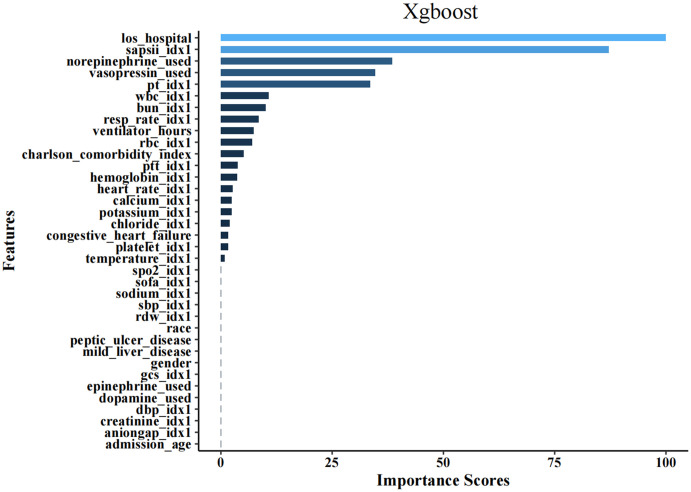
The top 36 features derived from XGBoost.

To clarify the significant features influencing model outputs, an analysis of feature importance was conducted. The top 36 features identified from the LR, SVM, Neural Network, and XGBoost models are presented in Fig 4. Within the XGBoost framework,hospital stay duration (los_hospital) and the SAPS II emerged as the most critical factors, followed by norepinephrine administration, vasopressin, PT, ventilator hours, WBC, and BUN levels (**Fig 5).** In the LR model, the SAPS II was recognized as the most significant predictor of in-hospital mortality; additionally, ventilator hours, length of stay, norepinephrine administration, vasopressin usage, PT, and WBC count also substantially contributed to mortality predictions (S1 Fig). Furthermore, in the SVM and Neural Network models, the SAPS II and ventilator hours were similarly identified as having considerable effects on the prediction of in-hospital mortality, as illustrated in S2 and S3 Fig.

### Interpretability analysis

**Fig 6:**
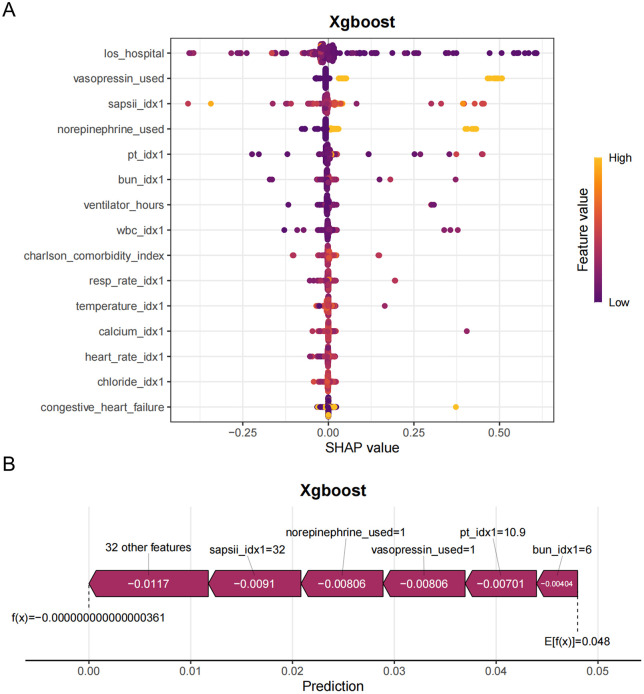
SHAP value distribution: Direction and magnitude of feature contributions.

To clarify the overall effects—both positive and negative—on model results and to investigate the similarities and differences in the significant characteristics of critically ill lung cancer patients across varying severity levels, the SHAP summary chart was employed. **Fig 6A** illustrates a detailed SHAP bee swarm plot that displays the impact of different features on the XGBoost model’s output.The horizontal axis represents the SHAP values, whereas the vertical axis ranks the features according to their cumulative effect on the SHAP values. Each data point corresponds to a unique instance, with its position along the x-axis reflecting the SHAP value associated with that specific instance and feature.

The plot clearly ranks the features by their overall impact on the model’s predictions. Features at the top of the plot (e.g., los_hospital, vasopressin_used, sapsii_idx1, norepinephrine_used) are the most important.Hospital stay duration(los_hospital) appears to be the most important feature. Most of the points are clustered around zero, but the color variance suggests a complex relationship to other variables. sapsii_idx1 is likely a severity score. It shows the patients with high severity scores (yellow dots) have a wider spread of shap values, which suggest a positive association.vasopressin_used and norepinephrine_used indicate the use of vasopressors, likely used to treat low blood pressure. Higher values of these features (yellow dots) tend to have positive SHAP values, suggesting that their use is associated with an increased predicted value.

As depicted in Fig 6B, S4 Fig sapsii_idx1 = 32 is a severity score. A value of 32 contributes −0.0091. the SAPS II score was identified as the most significant feature among the top 15 in the XGBoost model; a higher SAPS II score correlates with an increased likelihood of in-hospital mortality. This finding highlights the necessity of prioritizing the SAPS II score when predicting in-hospital mortality rates.All the features shown in the graph pushes the prediction to the left, meaning that each of them contributed to decreasing the value from the average to the final prediction value.We have added a correlation heatmap and a table of correlation coefficients for the top features (SAPS II, hospital stay duration, PT, and norepinephrine use) in the S5 Fig.Our analysis shows SAPS II shows almost no correlation with hospital stay duration(0.0778) and do not appear to pose a risk to the interpretability of the model.

## Discussion

In contemporary healthcare, the accurate prediction of in – hospital mortality for intensive care unit (ICU) patients with advanced lung cancer is of paramount importance. This study, leveraging the rich data within the Medical Information Mart for Intensive Care – IV (MIMIC – IV) database, aimed to develop and validate machine learning (ML) models for this purpose. Our findings contribute significantly to the existing body of knowledge, offering new insights into the factors influencing in – hospital mortality and highlighting the potential of ML in enhancing clinical decision – making.

Lung cancer patients admitted to ICUs exhibit significantly higher mortality rates than those with other solid malignancies, with results varying considerably by disease stage. Existing literature indicates mortality rates exceeding 50% for patients with advanced or extensive-stage disease. Specifically, Park et al. documented a 58.3% mortality rate among critically ill patients with advanced lung cancer in their Korean cohort study (2008–2010) [18]. Similarly, Song et al. reported mortality rates of 82.4% and 65.9% for stage IIIB/IV NSCLC and extensive-stage SCLC patients prior to and following 2011, respectively [19]. Our findings are more consistent with Adam et al.’s report of a 20% mortality rate in stage I NSCLC patients, likely attributable to our cohort’s predominance of primary rather than metastatic lung cancer cases [20].

This study developed a predictive model for in-hospital mortality among lung cancer patients using ML approaches. Twenty-seven clinical variables collected within the first 24 hours of ICU admission were analyzed using eight different ML algorithms. The XGBoost model exhibited superior performance, demonstrating robust discrimination, calibration, and significant clinical utility. Validation in an independent test cohort further affirmed the model’s reliability and accuracy. To enhance interpretability, we employed the SHAP framework for feature importance analysis. The results identified eight key predictors:hospital stay duration, SAPS II score, administration of norepinephrine or vasopressin, PT, duration of mechanical ventilation, WBC, and BUN levels. Additionally, SHAP force plots offered valuable insights into the model’s decision-making process for assessing individual patient mortality risk.

The XGBoost algorithm, an advanced implementation of gradient-boosted decision trees, demonstrates significant efficacy in handling large-scale datasets characterized by complex feature spaces. Medical research has widely adopted XGBoost-based predictive frameworks, revealing superior performance across various clinical domains, including sepsis management, cardiovascular risk assessment, and renal impairment evaluation [21–23]. Unlike traditional LR techniques, XGBoost effectively identifies non-linear associations and integrates multiple weak classifiers into a robust predictive model, enhancing generalizability. The SHAP framework offers valuable insights into ML model behavior by quantifying feature contributions, thereby improving model interpretability.The XGBoost model achieved the highest predictive performance, achieving an accuracy of 0.783, an F1 score of 0.595, and an AUC of 0.865 (95% CI: 0.840–0.891)within the training cohort. The performance metrics for the test cohort reflected similar trends, with an accuracy of 0.719, an F1 score of 0.543, and an AUC of 0.790(95% CI: 0.741–0.840).

This study systematically evaluated eight different ML approaches (LR, SVM, GBM, neural network, XGBoost, KNN, AdaBoost, and RF) for developing predictive models. Model performance was rigorously assessed using six established evaluation metrics: receiver operating characteristic curve analysis, F1 score, classification accuracy, sensitivity, specificity, and precision. Notably, our findings revealed superior predictive capability and stability with an ensemble approach that incorporated SVM, neural network, LR, and XGBoost, consistent with existing literature [24].

While comparing eight ML algorithms strengthens the methodological rigor of this study, the calibration curves (Fig A) reveal potential overfitting in some models, particularly Random Forest (RF) and k-Nearest Neighbors (k-NN).These models demonstrate a significant deviation from the ideal calibration line, suggesting that their predicted probabilities do not accurately reflect the observed event percentages in the validation dataset. Specifically, the observed event percentage is significantly higher in the validation data than the predicted probability from the KNN model and the RF model. This overconfidence in predictions is characteristic of overfitting, where the model has learned the training data too well and struggles to generalize to unseen data. While GBM also shows some calibration issues, the divergence is less pronounced than in RF and k-NN. This observation is reinforced when contrasting Fig 4A (training set) with Fig 4C (test set), where all models exhibit better calibration.

Through SHAP value analysis, we identified key predictive features, including hospital stay duration, SAPS II score, vasopressor administration (norepinephrine and vasopressin), PT, mechanical ventilation duration, WBC, and BUN levels. The SAPS II scoring system, a validated measure of organ dysfunction severity upon ICU admission, has demonstrated robust performance in mortality risk stratification [21,25–27]. Although extensively utilized for general ICU mortality prediction, the specific association between SAPS II scores and in-hospital mortality among lung cancer patients remains inadequately explored in current literature. Comparative studies have established the superior predictive validity of both SAPS II and SOFA scores for infection-related mortality in critical care populations, with score elevation correlating positively with increased mortality risk [28].

The association between the duration of ICU stay and patient mortality exhibits a biphasic pattern, indicating that both short stays (reflecting rapid clinical deterioration) and prolonged stays (suggesting persistent organ failure) are associated with increased mortality risks. However, existing research has not established precise cutoff values for these critical time points. Optimal clinical management should incorporate sequential physiological evaluations using standardized scoring systems, such as SAPS II, to facilitate comprehensive patient assessment [29,30]. If length of stay (LOS) is not known at admission, the use of first-day LOS can be explicitly stated as a proxy for SAPS II scoring.

Our predictive model exhibited consistent performance during external validation, confirming its robust generalizability across various clinical environments. Moreover, the selected predictive variables comprise standard clinical measurements, ensuring both ease of assessment and seamless integration across institutions with varying resource capacities.

Several methodological limitations merit attention in this study. First, the retrospective observational design introduces inherent selection bias and potential information bias due to inconsistencies in data collection and incomplete records. Second, whereas utilizing the MIMIC-IV database, the absence of external validation cohorts raises concerns regarding potential model overfitting, highlighting the necessity for future validation studies. Third, despite internal validation, the single-center nature of our data highlights the need for multicenter prospective studies to evaluate model generalizability across diverse populations. Most notably, the lack of histopathological and radiological characterization of lung cancer subtypes, particularly the distinction between small cell and non-small cell carcinomas, represents a significant limitation, as the absence of molecular diagnostic data may have affected model performance. Despite these limitations, our predictive model demonstrates clinical utility in assisting physicians with timely patient risk stratification.

In the process of developing our predictive model, we are acutely aware that several important clinical variables, namely cancer stage, metastasis, histology, and performance status, were not incorporated into the analysis. The absence of these variables has significant implications for the model’s clinical applicability.we acknowledge that the absence of these important clinical variables is a limitation of our study. By discussing the potential confounding effects and reasons for their missingness, we hope to provide a more comprehensive understanding of the model’s limitations and guide future research efforts to improve its clinical applicability.

While our XGBoost model demonstrates robust predictive accuracy in the MIMIC-IV cohort, its deployment in real-world ICU settings requires addressing several practical barriers. First, interoperability with existing electronic health record (EHR) systems is critical; this could be achieved through API-based integration or modular deployment within clinical decision support platforms.Second, clinician adoption hinges on interpretability—a challenge we mitigated via SHAP analysis (Fig 6), which quantifies and visualizes the impact of key variables (e.g., SAPS II, vasopressin use) on predictions. To foster trust, future iterations could incorporate interactive interfaces allowing clinicians to adjust inputs and observe risk estimates in real time. Regulatory approval would require prospective trials to evaluate model performance under dynamic ICU conditions and diverse patient demographics. A critical future direction involves deploying this model within a dedicated clinical platform to rigorously evaluate its runtime performance and integration feasibility.

## Conclusion

In a clinical setting, especially at the bedside, healthcare providers need quick and easy – to – use tools for risk assessment. While the XGBoost model demonstrated the best performance in predicting in – hospital mortality among critically ill lung cancer patients, it may be complex for immediate use in a busy ICU environment. A simplified risk score can distill the key information from the model into a more accessible format, enabling rapid decision – making.

Based on this study, multiple models (logistic regression, support vector machine, artificial neural network, and XGBoost) have consistently identified several key predictive factors, which can serve as the basis for a simplified clinical risk score. These factors include hospital stay duration, SAPS II scores, vasopressor administration (norepinephrine and vasopressin), coagulation parameters (PT), mechanical ventilation duration, and metabolic markers (WBC and BUN). The process of developing a simplified score includes determining the weight of each predictive factor. This can be based on the results of SHAP analysis, the application of SHAP analysis improved clinical interpretability by clarifying feature contributions to mortality risk, providing intensivists with actionable insights for prognostic assessment in critical care settings.

In conclusion, developing a simplified clinical risk score based on the key predictive factors identified in this study is expected to improve the bedside applicability of the model and support clinical decision – making in the ICU. Although, a notable limitation of our study is that the analysis solely relied on internal divisions from MIMIC – IV. This restricts the model’s applicability in broader clinical scenarios.

## Supporting information

S1 Fig The top 36 features derived from Logistic. (TIF)

S2 Fig The top 36 features derived from SVM. (TIF)

S3 Fig The top 36 features derived from Neural Network. (TIF)

S4 Fig Visually XGBoost model using SHAP. (TIF)

S5 Fig Correlation heatmap and a table of correlation coefficients for the top features (SAPS II, hospital stay duration, PT, and norepinephrine use). (TIF)
