# Supplementary figures and images for: Assessing in-hospital mortality risk in ICU lung cancer patients using machine learning: An analysis based on the MIMIC-IV database
Source: PLoS One. 2026 Jan 22;21(1):e0341259. doi: 10.1371/journal.pone.0341259 (PMC12826459; doi:10.1371/journal.pone.0341259)

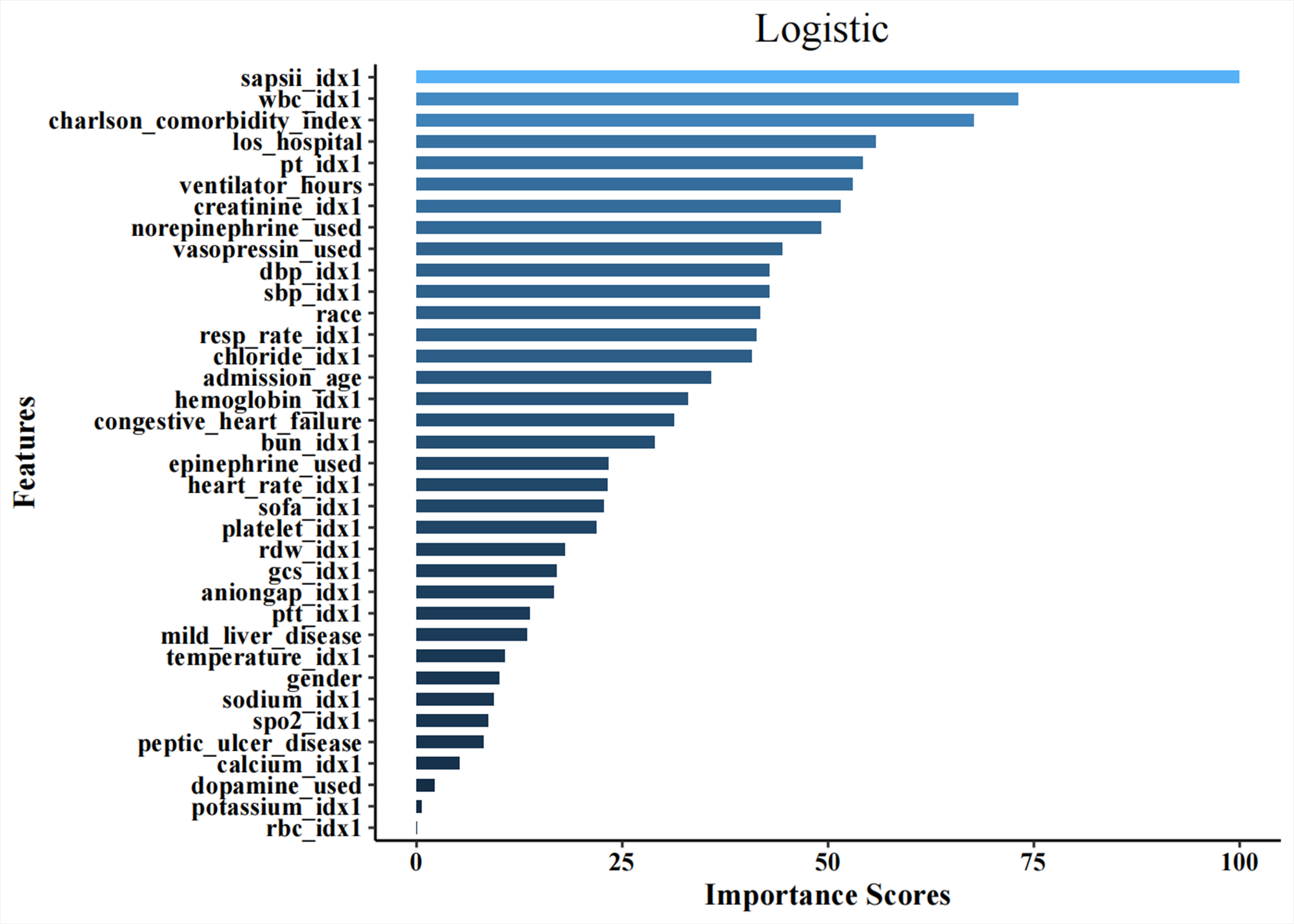

Supplement: S1 Fig — (TIF) [file pone.0341259.s001.tif]

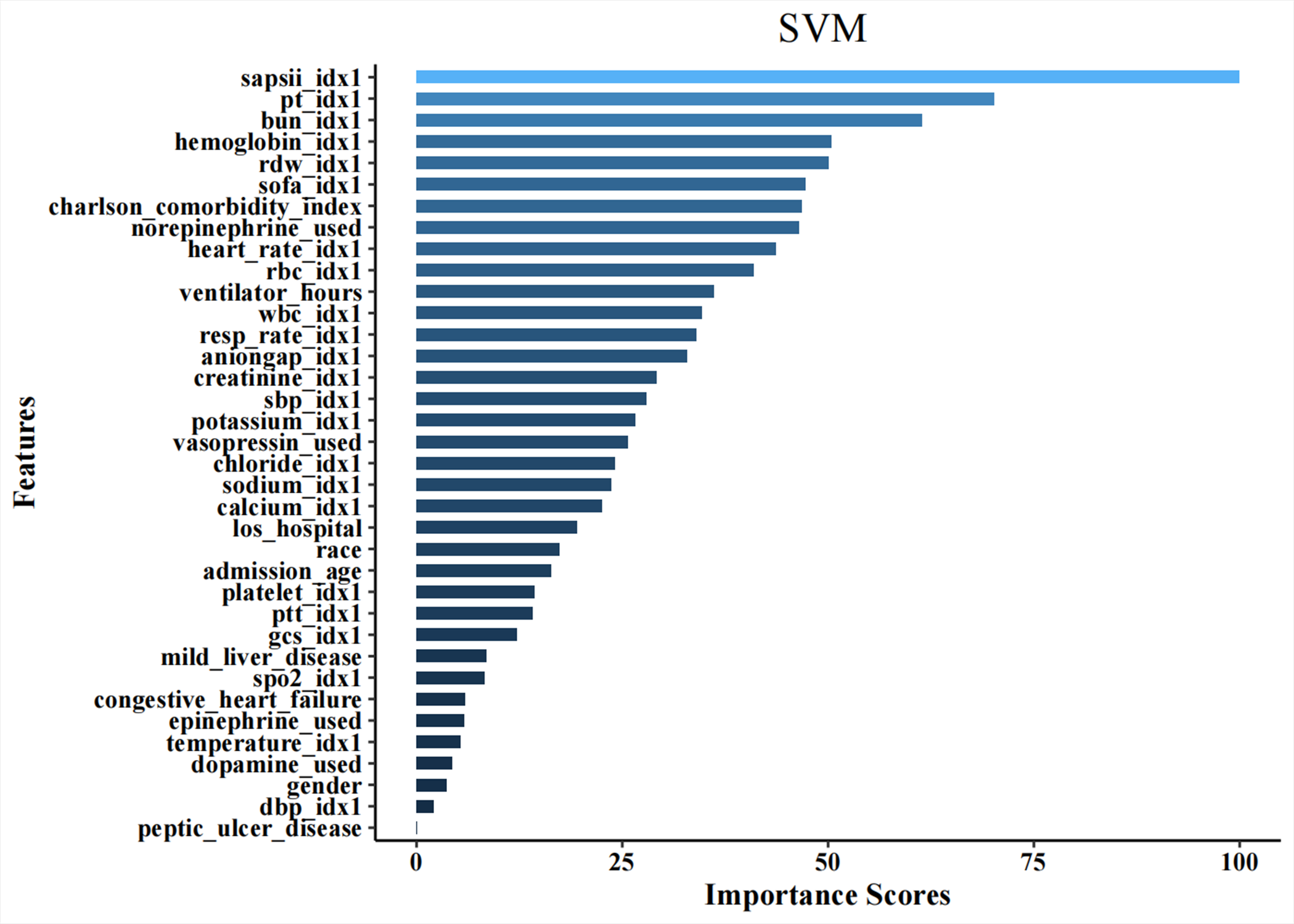

Supplement: S2 Fig — (TIF) [file pone.0341259.s002.tif]

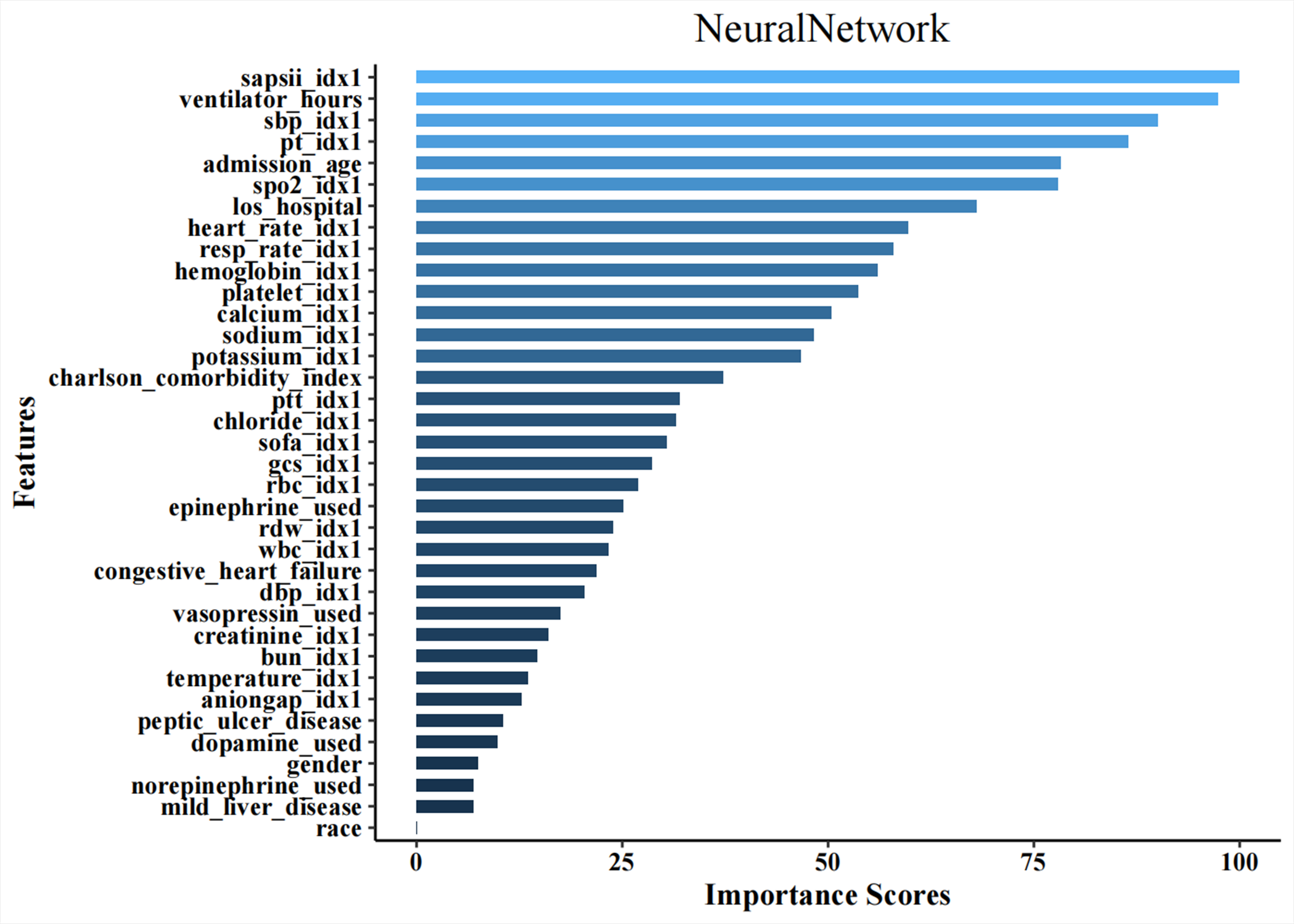

Supplement: S3 Fig — (TIF) [file pone.0341259.s003.tif]

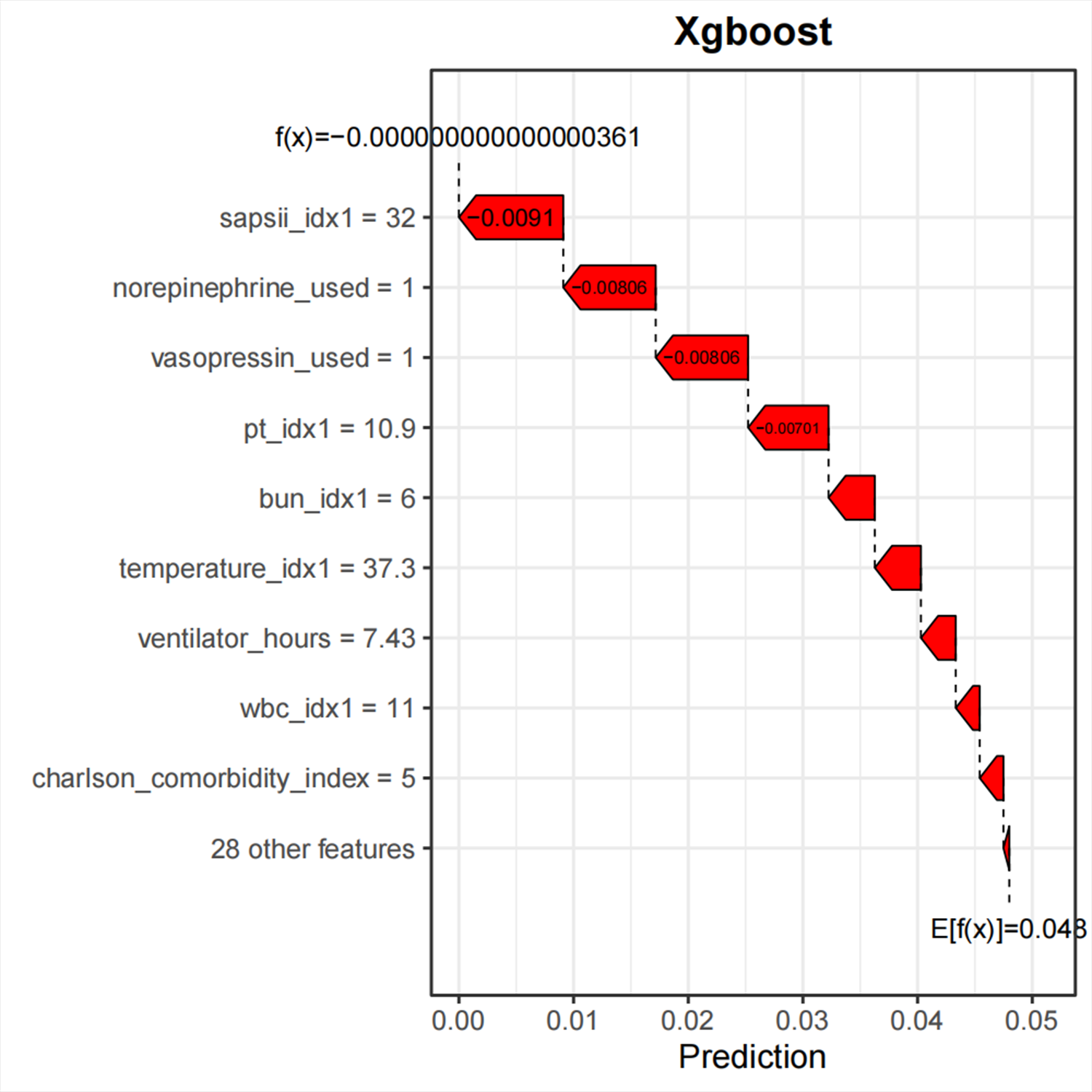

Supplement: S4 Fig — (TIF) [file pone.0341259.s004.tif]

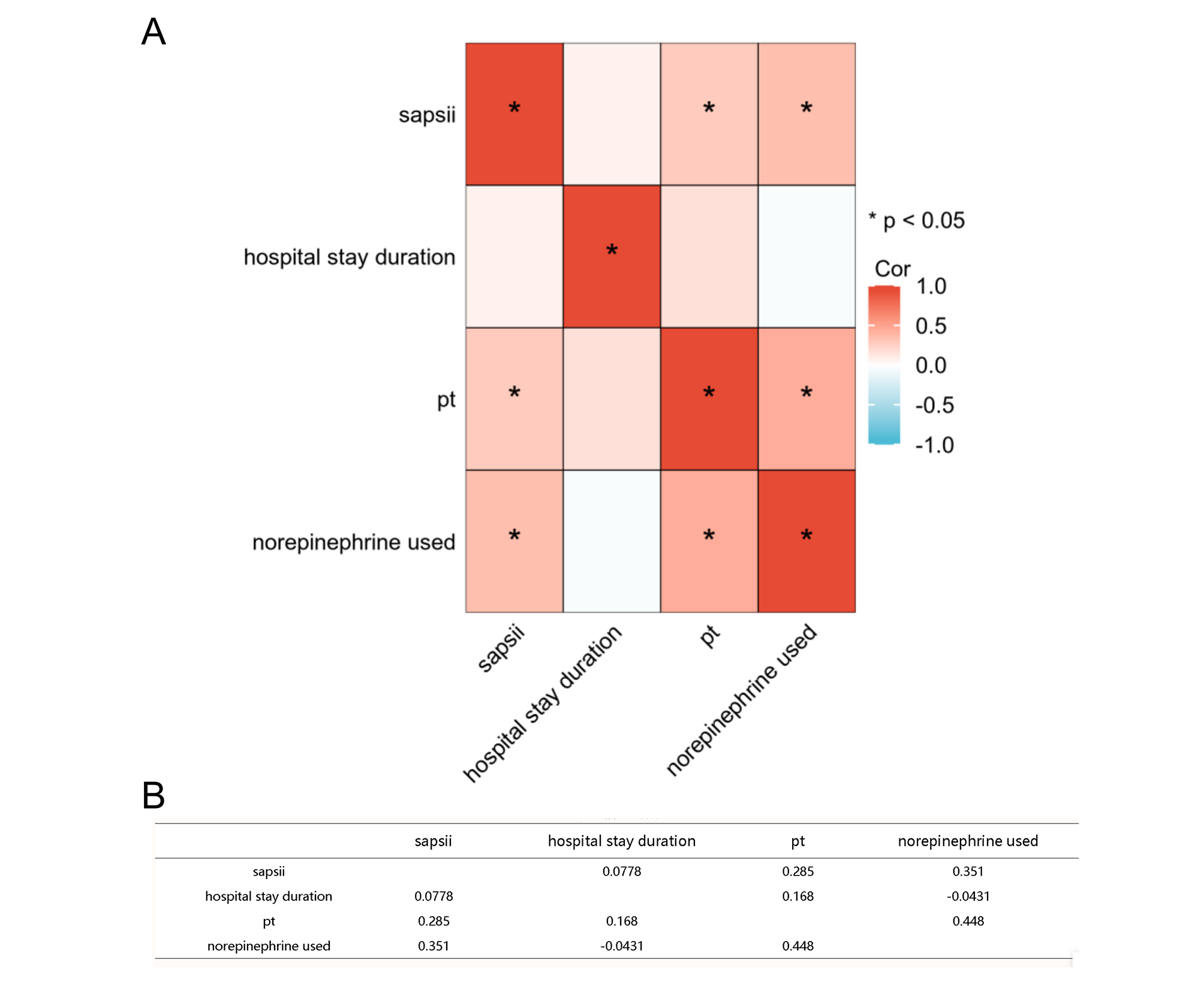

Supplement: S5 Fig — (TIF) [file pone.0341259.s005.tif]
